# Downregulation of salivary miR‐3928 as a potential biomarker in patients with oral squamous cell carcinoma and oral lichen planus

**DOI:** 10.1002/cre2.877

**Published:** 2024-03-13

**Authors:** Alieh Farshbaf, Farnaz Mohajertehran, Seyed Hamid Aghaee‐Bakhtiari, Hossein Ayatollahi, Katayoun Douzandeh, Atessa Pakfetrat, Nooshin Mohtasham

**Affiliations:** ^1^ Dental Research Center Mashhad University of Medical Sciences Mashhad Iran; ^2^ Oral and Maxillofacial Diseases Research Center Mashhad University of Medical Sciences Mashhad Iran; ^3^ Department of Oral and Maxillofacial Pathology, School of Dentistry Mashhad University of Medical Sciences Mashhad Iran; ^4^ Department of Medical Biotechnology and Nanotechnology, Faculty of Medicine Mashhad University of Medical Sciences Mashhad Iran; ^5^ Bioinformatics Research Center Mashhad University of Medical Sciences Mashhad Iran; ^6^ Hematology Department, Faculty of Medicine Mashhad University of Medical Science Mashhad Iran; ^7^ Pathology Department, Cancer Molecular Pathology Research Center Mashhad University of Medical Sciences Mashhad Iran; ^8^ Student Research Committee Mashhad University of Medical Sciences Mashhad Iran

**Keywords:** head and neck squamous cell carcinoma, miR‐3928, oral cancer, oral squamous cell carcinoma

## Abstract

**Objectives:**

Recent studies highlighted the role of miR expressed in saliva as reliable diagnostic and prognostic tools in the long‐term monitoring of cancer processes such as oral squamous carcinoma (OSCC). Based on a few previous studies, it seems the miR‐3928 can be considered a master regulator in carcinogenesis, and it can be therapeutically exploited. This is the first study that compared oral potentially malignant disorder (OLP) and malignant (OSCC) lesions for miR‐3928 expression.

**Materials and Methods:**

In this cross‐sectional study, saliva samples from 30 healthy control individuals, 30 patients with erosive/atrophic oral lichen planus, and 31 patients with OSCC were collected. The evaluation of miR‐3928 expression by q‐PCR and its correlation with clinicopathological indices were analyzed by Shapiro−Wilk, Kruskal−Wallis, Pearson's *χ* ^2^, and Mann−Whitney tests. The *p*‐value less than .05 indicated statistically significant results.

**Results:**

Based on nonparametric Kruskal−Wallis test results, there was a statistically significant difference between the ages of three study groups (*p* < .05). This test demonstrated a statistically significant difference between the average of miR‐3928 expression in three study groups (*p* < .05). The result of the *χ* ^2^ test showed a statistically significant difference in miR‐3928 expression between patients with OLP (*p* = .01) and also patients with OSCC (*p* < .0001) in comparison to the control group.

**Conclusions:**

The salivary miR‐3928 can play a tumor suppressive role in the pathobiology of OSCC, and it is significantly downregulated in patients. According to the potential tumor suppressive role of miR‐3928 in the OSCC process, we can consider this microRNA as a biomarker for future early diagnosis, screening, and potential target therapy.

## 1 INTRODUCTION

Head and neck squamous cell carcinoma (HNSCC) is introduced as the sixth most common cancer around the world, comprising 1%−4% of all cancers. The HNSCC represents different loco regions of the oral cavity, lip area, and upper aero digestive tract and more than 90% of them are oral squamous cell carcinoma (OSCC) (Bugshan & Farooq, 2020). Although chemoradiotherapy, surgery, photodynamic therapy, and using EGFR and COX‐2 inhibitors are applied for patients with OSCC, they showed relapse potential and metastasis. Approximately, one‐third of OSCC patients demonstrated recurrence, metastasis, and drug resistance, and 5‐year survival rate was poorly restricted to 50% (Ling et al., 2021). Early diagnosis and appropriate therapy can increase the 5‐year survival rate of OSCC by more than 90% and decrease mortality and morbidity rates (Radhika et al., 2016). The oral lichen planus (OLP) is a chronic autoimmune disease that is present in mucocutaneous sites. OLP is one of the oral potentially malignant disorders (OPMDs) originating from oral mucosal disorders. It is estimated that 1.4% of oral cavity lesions can transform into malignant lesions during 7 years. Besides the risk factors, OLP demonstrated malignancy potential to OSCC. It was reported erosive/atrophic type of OLP, female sex, and tongue location are more potent for malignant transformation (Giuliani et al., 2019; Hamour et al., 2020). The defined diagnostic criteria such as reliable biomarkers besides the histopathological confirmation can facilitate disease management and prevent improvement in the cancer process and epithelial dysplasia (González‐Moles et al., 2021).

Smoking, alcohol consumption, and HPV infection are known as the main lifestyle‐related risk factors in HNSCC pathogenesis. They can affect genetic profiles by alternation in the expression of proto‐oncogene or tumor suppressor genes. Therewith, the gene expression can be modified posttranscriptionally by microRNAs (miRNAs). The miRNAs are small noncoding RNAs (21−23 nt) that regulate gene expression by binding to the 3′ untranslated region (3′UTR) of messenger RNA (mRNA); they usually inhibit translation by degrading or inhibiting expression of target mRNAs. In some cases, miRNAs can attach to 5′UTR such as promoter and coding sequence and stimulate translation or gene silencing, respectively (Ali Syeda et al., 2020; Farshbaf, Mohtasham, et al., 2021). Related factors for this dynamic action of miRNAs are mRNA affinity, accessibility, and abundance of miRNAs and their target mRNA, mRNA secondary structures, 3′ UTRs binding, and ubiquitin proteins (O'Brien et al., 2018). In this study, we applied saliva samples because of cheap, noninvasive, and easy collection. Moreover, recent studies highlighted the role of miR expressed in saliva as a reliable diagnostic and prognostic tool in the long‐term monitoring of cancer processes such as OSCC (Farshbaf, Zare, et al., 2021; Roi et al., 2020). There are few studies about the miR‐3928 function, but the first study related to cancer discovered tumor suppressive role of miR‐3928 in osteosarcoma by targeting genes that participate in the bone tissue growth, cell cycle arrest, and immune system including ERBB3, IL‐6R, and CDK6 (Xu et al., 2014). Furthermore, exogenous overexpression of miR‐3928 in glioblastoma inhibited the expression of oncogenes including MDM2, CD44, DDX3X, HMGA2, CCND1, BRAF, ATOH8, and BMI1 and upregulated p53 phosphorylation in mice (Mulcahy et al., 2022). Based on the mentioned studies, it seems the miR‐3928 can be considered a master regulator in carcinogenesis, and it can be therapeutically exploited. Moreover, assessment of specific microRNA expression such as miR‐3928 with tumor suppressive role can be valuable in early diagnosis. In the present study, we evaluated the miR‐3928 expression in the OLP and OSCC groups in comparison to healthy controls. This is the first study that compares an OPMDs and malignant lesions for miR‐3928 expression.

## 2 METHODS AND MATERIALS

### 2.1 Study participants

Total fasting saliva samples including 30 healthy control individuals, 30 erosive/atrophic lichen planus, and 31 OSCC samples were obtained from the oncology section of Imam Reza, Omid and Quaem Hospitals, Department of Oral and Maxillofacial Pathology, School of Dentistry, Mashhad University of Medical Sciences, Mashhad, Iran from 2021 to 2022. The Mashhad University Ethics Committee (IR.MUMS.DENTISTERY.REC.1399.167) confirmed the research process. The informed consent forms were obtained from all participants in this research study. The fasting saliva samples were collected in sterile DNase and RNase‐free cryotubes and immediately transferred to an ice‐dried pack. Then, they were frozen at −80°C until RNA extraction.

The inclusion criteria were patients with OLP and OSCC who did not undertake antitumor therapy previously and did not report any other malignancy in their medical history, and in the control groups, the participants were without any systematic or inflammation diseases. Also, the saliva samples were with sufficient quality (without blood clots or mucous secretions) and quantity (≥300 µL saliva). The exclusion criteria were the samples without inclusion criteria and adequate quality or quantity and also the samples that pathologists did not approve appropriate criteria for patients with OSCC (Brierley et al., 2017) due to microscopic diagnosis.

### 2.2 RNA extraction and complementary DNA (cDNA) synthesis

We used RNX‐plus (SinaClon) reagent and modified the protocol instruction for saliva samples (Patel et al., 2011; Urbizu et al., 2023). First, 800 µL RNX‐plus was added to 300−500 µL obtained saliva samples and vortexed for 15 s to homogenize. Next, we incubated them at room temperature for 3−4 min. Then, 200 μL of chloroform (Merck Co.) was added to each tube vortexed for 15 s and incubated at room temperature for 3−5 min. The samples were then centrifuged at 12,000 rpm and 4°C for 20 min. Approximately, 500 μL of the upper aqueous layer from each sample was carefully transferred into new 1.5 mL DNase and RNase‐free microtubes. We repeated this step. Next, 500 μL of isopropanol (Merck Co.) was added to each microtube for RNA precipitation, and the microtubes were upside down for a few seconds. The microtubes were incubated overnight at −20°C. After the mentioned time, the samples were centrifuged at 12,000 rpm and 4°C for 45 min. The supernatant was discarded, and the observed pellet was washed with 1 mL of cold 80% molecular‐grade ethanol and centrifuged at 12,000 rpm and 4°C for 20 min. This step was performed twice. The pellet was air‐dried at room temperature for 3−4 min. Afterward, the pellet was resuspended in 20 μL of DEPC water and incubated at room temperature for 5 min. Finally, we used a quick spin to collect the sample at the bottom of the microtubes.

To determine the quantification and purity of extracted RNA, we used the Nanodrop system (Thermo Fisher Scientific 2000) to assess the ratio of wavelength absorbance in 260/280 nm. The RNAs with sufficient quantification (100 ng/µL) and purity (1.5−2 ratio) were utilized for cDNA synthesis. According to the manufacturer's standard protocol, the Adscript cDNAs synthesis Kit (REF: 22701; Bio‐Tech; addbio) was applied. In total 20 μL volume, 10 μL of 2x reaction buffer, 2 μL of 10 mM dNTP mixture, 1 μL 20x AddScript enzyme solution, 5 μL of RNA, 1 μL DEPC water, and 1 μL of 1 pM reverse transcription (RT) primer for U6 or miR‐3928 were mixed. The sequence of RT U6 primers were 5′‐GTCGTATGCAGAGCAGGGTCCGAGGTATTCGCACTGCATACGACAAAATATGG‐3′ and 5′‐GTCGTATGCAGAGCAGGGTCCGAGGTATTCGCACTGCATACGACGCCGAA‐3′ for miR‐3928. For each sample, the cDNA was synthesized separately by RT U6 and miR‐3928 primers. The temperature cycling was according to the manufacturer's protocol: preincubation at 25°C for 10 min, RT at 50°C for 60 min, RT inactivation at 80°C for 5 min, and holding at 12°C.

### 2.3 Quantitative real‐time PCR (qPCR)

Our real‐time qPCR reaction mixture contained 25 μL total volume for each reaction including 12.5 μL SYBR Green master mix (TQ110; SMOBIO), 2 μL cDNA, 3.5 μL distilled sterile water, and 1 μL of each 10 pm primers: miR‐3928 (F 5′‐TGGAGGAACCTTGGAGC‐3′), U6 (F 5′‐AAGGATGACACGCAAATTC‐3′) and universal (R 5′‐GAGCAGGGTCCGAGGT‐3′). Duplication in amplification was performed for all samples. The qPCR temperature process in Light Cycler 96 (Roche) was a preincubation step at 95°C for 30 s and 50 cycles at 95°C for 10 s and 60°C for 45 s. The ΔΔ*C*_t_ method was applied to measure the relative quantitation in gene expression. Moreover, we performed the analysis of the melting curve to confirm specific target amplification.

### 2.4 Data analysis

SPSS software (version 22) was applied for statistical analysis. The Shapiro−Wilk test was used for the assessment of normal distribution. The Kruskal−Wallis test was applied for the comparison of mean age (by year) in study groups, miR‐3928 expression between men and women according to the studied groups, and comparison of quantitative miR‐3928 expression in patients with OSCC by grade and also between OSCC (by grade) and control groups. The comparison of quantitative miR‐3928 expression in patients with OSCC by stage was evaluated by the Mann−Whitney test. Correlation between gender and studied groups and the comparison of quantitative miR‐3928 expression between study groups and control was applied by *χ* ^2^ test. The *p*‐value less than .05 indicated statistically significant results.

## 3 RESULTS

**Figure 1:**
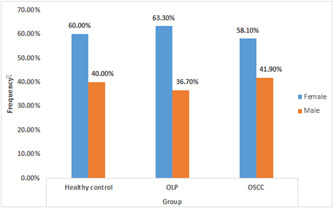
Distribution of sex by percent (%) in healthy control, OLP, and OSCC groups. OLP, oral lichen planus; OSCC, oral squamous carcinoma.

In Table 1, we presented demography information of study participants including age, sex, grade, and stage. The result of the Shapiro−Wilk test demonstrated that both age and miR‐3928 expression parameters were not normally distributed. Based on nonparametric Kruskal−Wallis test results, there was a statistically significant difference between the age of three study groups (*p* < .05) (Table 2). According to the *χ* ^2^ test, there was no statistically significant difference between the sex of the three study groups (*p* > .05), and all three groups were gender homogeneous (Figure 1). The nonparametric Kruskal−Wallis test demonstrated a statistically significant difference between an average of miR‐3928 expression in three study groups (*p* < .05) (Table 3).

**Table 1:** Registered demographic information of study participants including sex, age, grade, and stage.

Variants		Group			Total *N* (%)
		Healthy control *N* (%)	OLP *N* (%)	OSCC *N* (%)	
Sex	Female	18 (60.0%)	19 (63.3%)	18 (58.1%)	55 (60.4%)
	Male	12 (40.0%)	11 (36.7%)	13 (41.9%)	36 (39.6%)
Grade	Grade I	‐‐‐	‐‐‐	17 (54.8%)	17 (54.8%)
	Grade II			10 (32.3%)	10 (32.3%)
	Grade III			4 (12.9%)	4 (12.9%)
Stage	Advanced	‐‐‐	‐‐‐	11 (35.5%)	11 (35.5%)
	Early			20 (64.5%)	20 (64.5%)
Age	<35 years old	20 (66.7%)	7 (23.3%)	3 (9.7%)	30 (33.0%)
	>35 years old	10 (33.3%)	23 (76.7%)	28 (90.3%)	61 (67.0%)

Abbreviations: OLP, oral lichen planus; OSCC, oral squamous carcinoma.

**Table 2:** Comparison of mean age (by year) in study groups.

Group	*N*	Mean ± standard deviation	Standard error	95% confidence interval for mean		Minimum	Maximum	Kruskal−Wallis test
				Lower bound	Upper bound			
Control	30	35.07 ± 11.095	2.026	30.92	39.21	24	66	*p* < .0001
OLP	30	49.10 ± 15.103	2.757	43.46	54.74	25	73	
OSCC	31	54.84 ± 12.464	2.239	50.27	59.41	28	78	
Total	91	46.43 ± 15.320	1.606	43.24	49.62	24	78	

Abbreviations: OLP, oral lichen planus; OSCC, oral squamous carcinoma.

**Table 3:** Comparison of average in miR‐3928 expression between study groups.

Group	*N*	Mean	Standard deviation	95% confidence interval for mean		Minimum	Maximum	Kruskal−Wallis test
				Lower bound	Upper bound			
Control	30	67.58	100.14	30.19	104.97	0.04	300.20	*p* < .0001
OLP	30	6.53	10.15	2.74	10.32	1.19	45.05	
OSCC	31	1.71	2.88	0.66	2.77	0.03	15.18	
Total	91	25.02	64.59	11.56	38.47	0.03	300.20	

Abbreviations: OLP, oral lichen planus; OSCC, oral squamous carcinoma.

**Figure 2:**
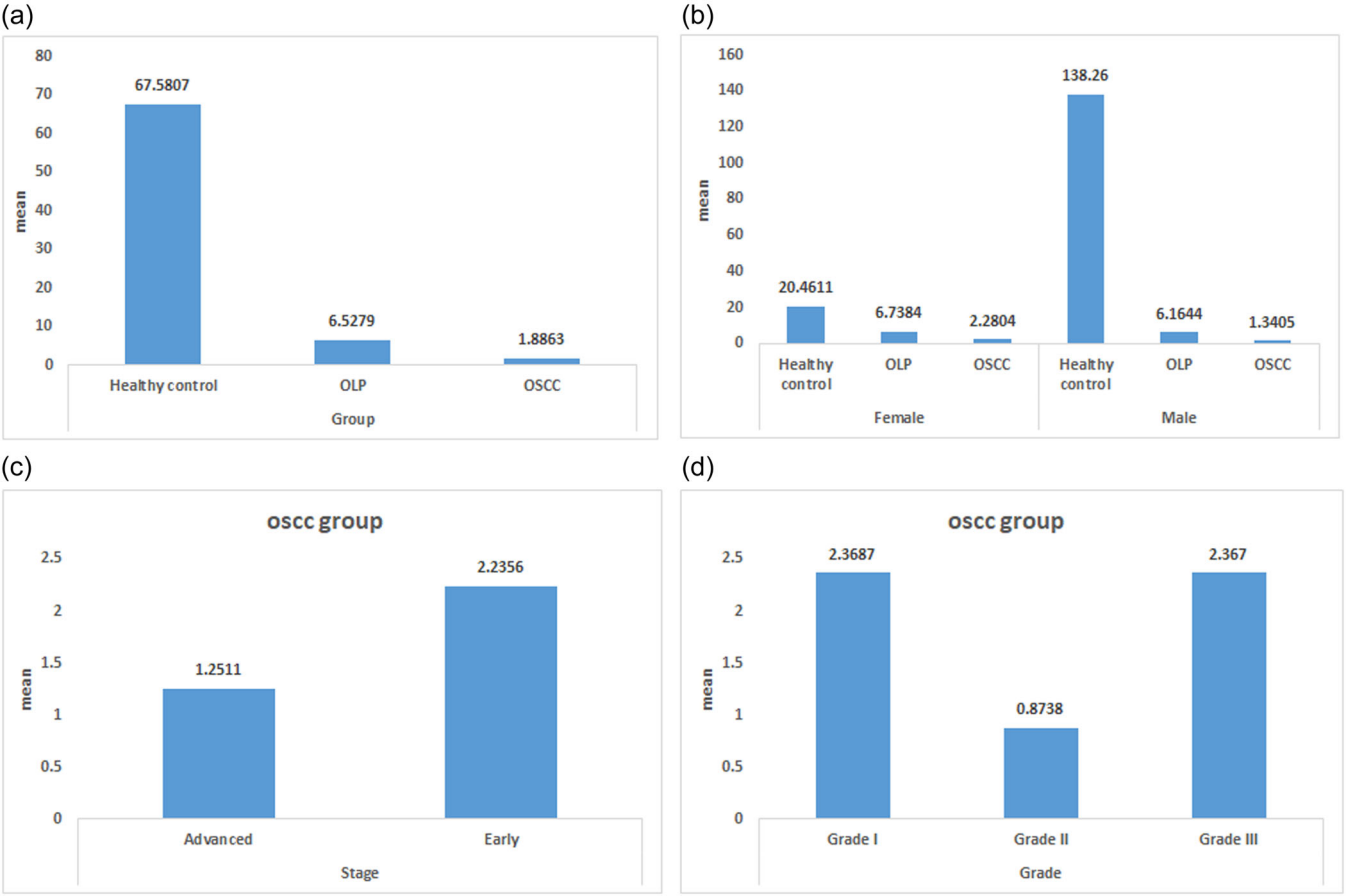
Comparison of the mean miR‐3928 expression's fold‐change between OSCC, OLP, and healthy control groups (a). Comparison of the mean of miR‐3928 expression by sex between OSCC, OLP, and healthy control groups (b). Comparison of the mean of miR‐3928 expression by stage (c) and grade (d) in the OSCC group. OLP, oral lichen planus; OSCC, oral squamous carcinoma.

The mean expression of miR‐3928 was significantly reduced in OSCC (67‐folds) and OLP (sixfolds) groups compared to healthy control (Figure 2a). The results of the Mann−Whitney test showed there were no statistically significant differences between the mean of miR‐3928 expression by sex and OSCC (*p* = .40) or OLP (*p* = .747) groups; however, there were statistically significant differences between the mean of miR‐3928 expression by sex and the healthy control group (*p* = .038) (Figure 2b). The results of the Mann−Whitney test showed there were no statistically significant differences between the mean of miR‐3928 expression early and advanced stage in the OSCC group (*p* = .186) (Figure 2c). The results of the Kruskal‐Wallis test demonstrated there were no statistically significant differences between the mean of miR‐3928 expression and different grades of the OSCC group (*p* = .365) (Figure 2d).

**Figure 3:**
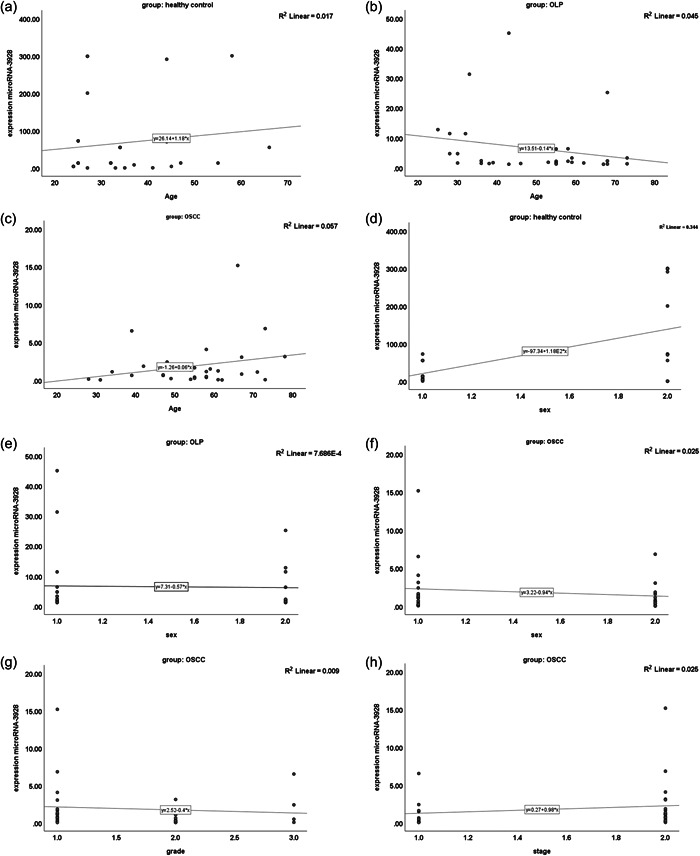
Correlation between miR‐3928 expression and clinical parameters including age (a−c) and sex (d−f) in healthy control, OLP, and OSCC groups, respectively. Correlation between miR‐3928 expression and grade (g) and stage (h) in the OSCC group. OLP, oral lichen planus; OSCC, oral squamous carcinoma.

There was no correlation between miR‐3928 expression and different clinical parameters including age (Figure 3a−c), and sex (Figure 3d−f) in healthy control, OLP, and OSCC groups, respectively. In addition, we showed there was no correlation between miR‐3928 expression and grade (Figure 3g) and stage (Figure 3h) in the OSCC group.

## 4 DISCUSSION

In this study, we evaluated the difference in miR‐3928 expression between a potentially malignant disorder (OLP) and malignant (OSCC) lesions in comparison to control. We showed the expression of miR‐3928 significantly reduced in OSCC (67‐folds) and OLP (sixfolds) groups in comparison to healthy controls. Based on our result, miR‐3928 can be considered a valuable biomarker and can be applied as a tumor inhibitor in therapeutic approaches. On the other hand, saliva is a reliable source for miR‐3928 assessment besides the substantial benefits of nature.

Data analysis of next‐generation sequencing discovered a significant difference in the expression of 638 miRNAs in oral cancer patients (*n* = 12) in comparison to healthy controls (*n* = 12). The oral cancer group showed reducing in 373 miRNAs and increasing in 265 miRNAs expression by qRT‐PCR, the miR‐3928 was among 27 detected miRNAs differentially expressed and associated with oral cancer (Fadhil et al., 2019). Following the results of this study, Fadhil et al. (2019) candidate five of these miRNAs for assessment, and one of them was miR‐3928. They demonstrated a significant difference in miR‐3928 expression by RT‐qPCR between patients with HNSCC (*n* = 150) and healthy controls (*n* = 80). The expression of miR‐3928 was strongly related to lymph node metastasis and tumor size (Fadhil et al., 2020). In contrast to their study, we evaluated the relationship between miR‐3928 expression and grade. Besides, we considered OSCC and erosive/atrophic OLP to evaluate specific malignant and premalignant oral lesions instead of different types of HNSCC. Mulcahy et al. (2022) reported a significant reduction in endogenous miR‐3928 expression by RT‐qPCR in patients with glioblastoma (*n* = 17) and glioblastoma (GBM) cell lines (*n* = 7). They overexpressed exogenous miR‐3928 in the four different GBM cell lines and discovered a notable reduction in proliferation and impeding cell growth. This was because of miR‐3928 function in increasing of factors involved in cell cycle G1/S arrest. The result of in vivo GBM xenograft (*n* = 5) by overexpressed miR‐3928 demonstrated significant repression in tumor volume. They approved the tumor‐suppressive role of overexpressed miR‐3928 which represses several oncogenes and upregulated p53 expression (Mulcahy et al., 2022).

The in vivo and in vitro study of Xu et al. in 2014 demonstrated downregulation of miR‐3928 expression in both patients with osteosarcoma (*n* = 10) and osteosarcoma cell lines (*n* = 3) in comparison to normal tissues and human osteoblast cell lines, respectively. They showed induction of miR‐3928 overexpression up to six to eightfolds after mimics' transfection in two cell lines that inhibited the proliferation and increased the apoptosis and the number of cells in the G1 phase. It improved cell growth. In contrast, downregulation of miR‐3928 expression improved cell proliferation. They guessed reduction of miR‐3928 levels prompted angiogenesis by increasing CDK6 expression (Xu et al., 2014). The results of the mentioned studies approved the tumor suppressive role of miR‐3928 in the cancer process. Based on previous studies, this valuable biomarker is involved in cell cycle and cellular differentiation of squamous cells, and miR‐3928 downregulation activates oncogenes. In this regard, it can apply to predictive prognosis and future therapeutic approaches.

To this date, there was no report to determine the miR‐3928 function in patients with OLP; however, Mehdipour et al. in 2023 demonstrated salivary overexpression of miR‐146a and miR‐155 in dysplastic OLP and OSCC; they suggested that it can be considered as a sign of beginning malignancy. Their results were obtained by comparing 60 patients including dysplastic OLP, non‐dysplastic OLP, OSCC, and healthy controls (*N* = 15 in each group) (Mehdipour et al., 2023). In addition, it was reported that down‐regulation of miR‐214, miR‐26a/b, and overexpression of miR‐27a/b, and miR‐146a and miR‐155 upregulated in OLP patients (Li et al., 2022). For the first time, Song et al. (2021) mapped the expression profile of alternatively expressed circular RNAs (circRNAs) in patients with OLP by comparing six OLPs with six normal oral mucosal tissues. They revealed 135 circRNAs differentially expressed in OLP 83 circRNAs upregulated and 52 circRNAs downregulated. Among them, 10 circRNAs changed significantly their expression in OLP that participated in 78 signal pathways (Song et al., 2021). Chen et al. in 2017 by analyzing miRNA‐mRNA networks showed a total of 94 differentially expressed miRNAs and 599 differentially expressed genes in OLP due to comparing nine patients with OLP to four healthy controls. Among them, five miRNAs downregulated including Hsa‐miR‐135a‐5p, hsa‐miR‐128‐3p, hsa‐miR‐218‐5p, has‐miR‐125a‐5p, and hsa‐let‐7e‐5 that can be considered as potentially biomarkers in patients with OLP (Chen et al., 2017).

Some of the miRNAs can act as onco‐miRNAs by the copy number amplification mechanism. The genome‐wide oncogenic onco‐miRNA screening of squamous cell carcinoma of the lung detected 231 miRNAs that altered their copy numbers. Among 11 highly expressed onco‐miRNAs, the three types are associated with poor prognosis, including miR‐296‐5p, miR‐324‐3p, and miR‐3928‐3p‐that suppressed FAM46C‐and induced MYC expressions and also improved carcinogenesis in SCC of the lung (Xia et al., 2018).

The TLRC‐m0008_3p is a variant of this miR (known as miR‐3928v) detected by solexa sequencing that is highly expressed in HBV^+^ human hepatocellular carcinoma (HCC) tissue (*n* = 20), as well as the high expression of miR‐3928v reported in the serum of patients with HCC (*n* = 60) as compared with healthy individuals (*n* = 30). The miR‐3928v plays an oncogenic role that improves cell viability, proliferation, and G1/S transition and also reduces cell apoptosis (Zhang et al., 2018). The miR‐3928v prompted metastasis and invasion by increasing vimentin, matrix metallopeptidase 2 (MMP2), and MMP9 expressions. The voltage‐dependent anion channel 3 (VDAC3) gene consists of a 3′ UTR binding site for miR‐3928v attachment that increases apoptosis and inhibits G1/S transition. Therefore, VDAC3 plays a tumor‐suppressive role; it is downregulated by miR‐3928v. HBx as a critical factor augments the miR‐3928v expression that is regulated by EGR1 in the upstream NF‐κB pathway; it improves HCC development (Zhang et al., 2018). The other variant of miR‐3928 (rs5997893) is reported to notably interact with HLA‐miRNA and is suggested to be considered for target therapy in rheumatoid arthritis (Guo et al., 2021).

Recent studies focused on the role of salivary miRNAs in the etiopathogenesis of OSCC. It demonstrated the tumor suppressive role of salivary miR‐15a and miR‐16‐1 according to their downregulation in patients with OSCC (*n* = 15) in comparison to healthy individuals (*n* = 15) (Koopaie et al., 2021). Salivary miR‐30c‐5p expression was significantly downregulated in patients with OSCC (*n* = 33) in comparison to healthy controls (*n* = 12) that target p53 and Wnt pathways in OSCC pathogenesis (Mehterov et al., 2021). The salivary microRNA‐21 is proposed as a biomarker for the prediction of cervical lymph node metastasis before surgery in patients with OSCC (*n* = 130) (Jadhav et al., 2022). It was suggested that salivary miR‐31 can be considered as a postoperative biomarker for follow‐up monitoring because the miR‐31 expression in preoperative patients with OSCC was significantly higher than that in postoperative patients (Kumari et al., 2021). For the first time, it was reported only miR‐423‐5p is an independent prognostic potential promising and diagnostic biomarker that is highly expressed in patients with OSCC (Romani et al., 2021). The three‐miRNA panel is introduced for OSCC discovery that identified salivary cancer‐specific biomarkers: miR‐31‐5p, miR‐345‐3p, and miR‐424‐3p are significantly upregulated in patients with OSCC and play onco‐miR roles (Scholtz et al., 2022). In the mentioned study, scientists believe the assessment of several miR expressions (as panel) is more reliable in the detection of cancer than one. The results of these studies hallmark the valuable role of salivary miRNAs as noninvasive biomarkers in prognosis and early diagnosis.

More investigation about master‐involved genes in OSCC pathogenesis provides the opportunity to detect biomarkers such as miRNAs for early diagnosis and therapeutic approaches. The result of bioinformatic analysis by Yang et al. in 2021 revealed that 261 genes dysregulated in the tissues of OSCC patients; 10 genes are known as hub genes including FN1, CXCL8, CXCL10, SPP1, FOXM1, AURKA, ISG15, PLAUR, TPX2, and BIRC5. The FOXM1 and TPX2 are proposed to be candidates for immunotherapy approaches, and several genes are suggested for the prediction of OSCC prognosis (Yang et al., 2021). Since one gene can bind to multiple miRNAs and one microRNA can be attached to several genes, it seems similar studies like Yang et al. are valuable to be continued for the identification of miRNAs that target previously detected genes. In future studies, we proposed the evaluation of individuals with genetic background for screening of a potentially malignant disorder such as OLP that is susceptible to OSCC, before clinical presentation and malignancy transformation. Since some variants of miR‐3928 play an oncogenic role in prognosis and therapeutic approaches, assessment of different miR‐3928 subsets is needed to ensure tumor suppressive functions. Moreover, we suggest more investigation into the correlation between autoimmune disease, HLA‐typing, and miR‐3928 in a large study population.

## 5 CONCLUSION

According to our result and previous studies based on the pivotal role of miR‐3928 in the regulation of genes that are involved in the pathogenesis of OSCC, we need more developing studies with multiple microRNA candidates as biomarkers for definitive early diagnosis, screening, prognosis, and potential target therapy. To achieve this, we can apply advanced technology for high‐throughput analysis before beginning therapy and screening tumor response behavior following the personalized difference for precision medicine. We showed that salivary miR‐3928 potentially plays a tumor suppressive role in the pathobiology of OSCC, and it is significantly downregulated in patients.

## AUTHOR CONTRIBUTIONS

Nooshin Mohtasham and Farnaz Mohajertehran contributed to the study's conception and design. Alieh Farshbaf and Nooshin Mohtasham contributed to the data acquisition, analysis, and interpretation. Alieh Farshbaf wrote the manuscript and Alieh Farshbaf and Nooshin Mohtasham revised the manuscript. SHossein Ayatollahi designed the sequence primers. Farnaz Mohajertehran, Hossein Ayatollahi, Katayoun Douzandeh, and Atessa Pakfetrat collected the blood samples and approved the defivitive diagnosis of patients. All authors reviewed and approved the final version of the manuscript.

## CONFLICT OF INTEREST STATEMENT

The authors declare no conflict of interest.

## Data Availability

The data that support the findings of this study are available from the corresponding author upon request.
